# A Brief History of Qinghaosu

**DOI:** 10.1016/j.pt.2015.10.010

**Published:** 2015-12

**Authors:** Nicholas J. White, Tran T. Hien, François H. Nosten

**Affiliations:** 1 Mahidol-Oxford Tropical Medicine Research Unit, Faculty of Tropical Medicine, Mahidol University, Bangkok, Thailand; 2 Centre for Tropical Medicine and Global Health, Nuffield Department of Medicine, Oxford University, Oxford, UK; 3 Oxford University Clinical Research Unit, Hospital for Tropical Diseases, Ho Chi Minh City, Viet Nam; 4 Shoklo Malaria Research Unit, Faculty of Tropical Medicine, Mahidol University, Tak, Thailand

## Abstract

The 2015 Nobel Prize for Medicine or Physiology was awarded to William C. Campbell and Satoshi Ōmura for their discovery of avermectins, and to Tu You You for her contribution to the discovery of artemisinin. The discovery and development of qinghaosu (artemisinin) as an antimalarial drug is a remarkable and convoluted tale.

In the mid-1960s, in the immediate aftermath of the Cultural Revolution, China responded to requests from North Vietnam for help in their impending conflict. Malaria had played a major role in the first and second World Wars, and it had nearly killed Ho Chi Minh in 1945. Ho knew malaria could be a decisive factor in the forthcoming struggle. Malaria was still a significant problem in China too and so scientists across the land were ordered to find effective remedies, both in modern pharmaceutical chemistry and also in the extensive traditional medicine pharmacopoeia. On the 23 of May 1967, Project 523 was formed. This remarkable truly multicentre collaboration discovered the antimalarial properties of organic extracts of the leaves of *Artemisia annua*, a traditional febrifuge, identified the antimalarial moieties, determined their chemical structures, and characterized their physico-chemical properties and their antimalarial activities, first in animal models, and then in human malaria [1]. Initially there was some confusion over the plant; was it qinghao or huanghuahao that had the magical antimalarial properties? Misidentification delayed proceedings but once the correct plant extract was used, it was clear that the active moiety (now called qinghaosu, or later–artemisinin) was an extremely active antimalarial. Indeed it produced the most rapid parasite clearance of any known antimalarial drugs. Skillful chemistry then produced the reduction derivative dihydroqinghaosu (dihydroartemisinin) which was even more potent, and this served as the basis for stable lipophilic and hydrophilic derivatives (artemether and artesunate, respectively). Led by Professor Li Guo Qiao, a professor of traditional Chinese medicine from Guangzhou, clinical trials were conducted which confirmed the extraordinary antimalarial activity of these compounds both in uncomplicated and cerebral malaria 2, 3, 4. In 1979 the Qinghaosu Antimalaria Coordinating Research Group published a remarkable succinct description (in English) of the physicochemical properties, antimalarial activity, and clinical evaluation of artemisinin in the *Chinese Medical Journal* [2] (Figure 1). Slowly, the news spread.

**Figure 1:**
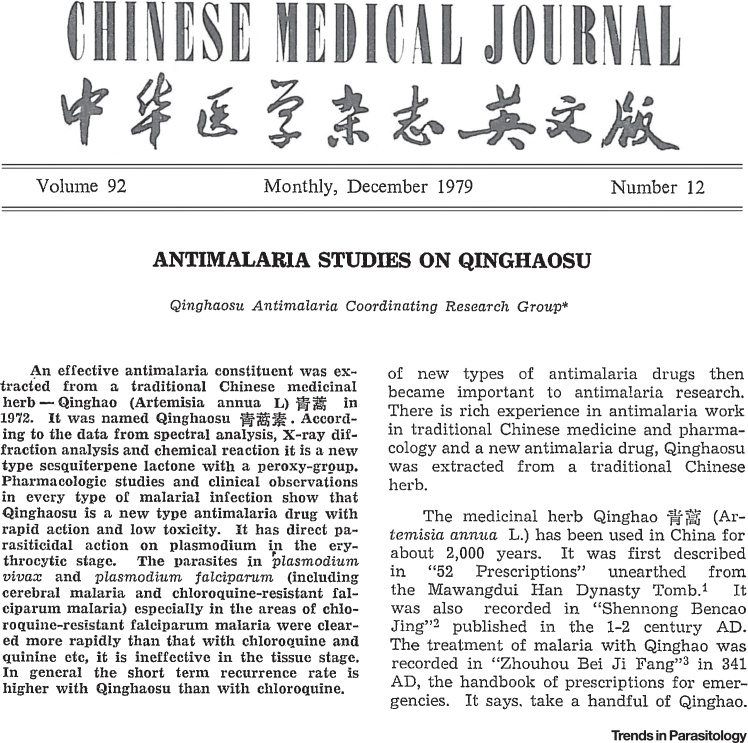
The Remarkable First Publication in English Describing the Discovery and Development of Qinghaosu.

Thereafter things went neither smoothly or quickly. The Chinese interaction with TDR, the World Health Organization (WHO) Special Programme for Research and Training in Tropical Diseases, which had a strong US Army representation at the time, was uneasy and ultimately fruitless. With dubious scientific justification, TDR decided to develop the ethyl ether (arteether) of dihydroartemisinin as a new drug, rather than the methyl ether (artemether) produced by a Chinese pharmaceutical company (the Chinese had actually synthesized arteether but rejected it in favour of artemether). Questions were raised over the quality of the Chinese drugs, and the stability of the water soluble artesunate. WHO TDR and the US Army decided initially to focus only on the intramuscular oil based injections and not to develop oral or intravenous drugs, which today form the mainstay of antimalarial treatment (http://apps.who.int/iris/bitstream/10665/61147/1/TDR_CHEMAL_ART_86.3.pdf). Meanwhile, antimalarial drug resistance continued to worsen in Southeast Asia. Effective alternatives treatments were needed desperately. Asian investigators, tired of waiting for the ‘quality’ products promised by WHO, began studies with Chinese oral, parenteral and rectal formulations in Myanmar, Vietnam (which by the late 1980s was producing its own artemisinin), Thailand 5, 6, 7 and soon after in Africa [8]. These rapidly confirmed the original Chinese claims.

The first large randomized controlled trials in severe malaria, which started in 1991, were with Chinese artemether [9]. These showed superiority in terms of mortality reduction in adults from Southeast Asia, but not in African children and left sufficient equipoise that quinine (another venerable plant derived compound) remained as the treatment of choice [10]. The oral drugs (artemisinin, artesunate, or artemether) were rapidly effective and well tolerated but as monotherapies they required treatment courses of 5 and 7 days in vivax and falciparum malaria, respectively. Combinations with more slowly eliminated antimalarial drugs proved highly effective and, eventually, 3-day treatments became established 4, 6, 7. The excellent tolerability and efficacy of the artemisinin-based combination therapies (ACTs) in Southeast Asia eventually led to confirmatory trials in South America and across Africa, which began in the late 1990s [7]. Although the artemisinin derivatives were very well tolerated as well as being rapidly effective there were two prevailing safety concerns at the time; first, repeated high injected doses of the oil based arteether (and artemether) caused an unusual pattern of selective neurotoxicity affecting certain brain stem nuclei in rodents and beagle dogs; second, artemisinins were embryotoxic. Fortunately, neurotoxicity was never confirmed in humans, but until recently artemisinins were contraindicated in the first trimester of pregnancy in uncomplicated malaria infections, although there is increasing evidence for safety in early pregnancy.

During the 1990s it became increasingly clear that the continued use of inexpensive, yet ineffective, antimalarial drugs (chloroquine and then sulphadoxine-pyrimethamine) by most malaria endemic countries was killing millions of people (most of whom were children in Africa). Meanwhile, the evidence that ACTs were highly effective and well tolerated had grown steadily. The consistently good results from Asia were replicated elsewhere and toxicity concerns receded 7, 8, 9, 10. The debate between malaria researchers, non-governmental organizations (NGOs), and growing numbers of malaria control programme representatives who argued strongly for deployment of these drugs, and the donors and international organisations who were reluctant to support them, became increasingly heated [11]. Finally in 2006, 27 years after the first seminal publication in English (Figure 1), the WHO decided clearly and unequivocally to recommend ACTs as first line treatment of uncomplicated falciparum malaria in all endemic countries [12]. At the same time, the WHO raised the bar substantially in the minimum efficacy required of an antimalarial treatment – malaria control programmes everywhere were now requested to aim for 28-day ‘cure’ rates of 95% and to change policy if cure rates fell below 90% [12]. Previously it had been considered acceptable for failure rates assessed at 14 days to be as high as 25% (which corresponded to true failure rates over 50%).

The rapid parasite clearance caused by the artemisinins and the associated speedy clinical recovery had long suggested that these compounds conferred a survival benefit in severe malaria. Unfortunately, because the oil-based intramuscular formulations (artemether, arteether) were then the compounds favoured by the WHO, these were the first to be evaluated in large randomized trials in severe malaria. The results were not sufficiently powerful to change practice [10], probably because these oil based intramuscular drugs are slowly and unreliably absorbed from the injection site. In contrast, the water-soluble artesunate can be given intravenously and is rapidly and reliably absorbed following intramuscular injection. Belatedly in 2003 multicentre randomized trials with parenteral artesunate began in Asia. These showed a substantial (35%) reduction in mortality compared with quinine [13]. This result was sufficient for policy change outside Africa, and it paved the way for the largest randomized controlled trial in African children hospitalised with severe malaria (AQUAMAT) [14]. The AQUAMAT trial showed a 22.5% lower mortality in children who received artesunate compared with those who received quinine. This coincided with removal of lingering concerns over drug quality and led to a uniform recommendation for parenteral artesunate as the treatment of choice for severe malaria everywhere.

In recent years substantial increases in international funding for malaria control have resulted in widespread deployment of ACTs in nearly all malaria endemic areas, and contributed to a substantial decline in global malaria morbidity and mortality (World Malaria Report 2014: http://www.who.int/malaria/publications/world_malaria_report_2014/en). Malaria elimination is again on the political agenda. Although there are formidable obstacles to this ambitious goal, it cannot be achieved without effective antimalarial medicines. In January 2006, the WHO recognized the risks of artemisinin resistance arising from decades of poorly regulated use and widespread availability of falsified and sub-standard medicines, and pushed strongly for a ban on monotherapies, but unfortunately, this was too late to prevent the emergence of resistance to artemisinin. Today artemisinin resistant *Plasmodium falciparum* can be found across Southeast Asia from the coast of Vietnam to the Myanmar–India border [15]. Predictably, uncontained resistance to artemisinin has led to worsening resistance to the ACT partner drugs. The prospect of drug resistant malaria parasites spreading from Southeast Asia through India to Africa and killing millions of children for a third time has rightly excited alarm, and provoked numerous meetings and resolutions, but it has not resulted in a radical containment strategy. For most of the malaria affected world, there is no evidence yet that the products of this remarkable Chinese traditional medicine are failing – but continued vigilance is needed. Loss of the artemisinins would deal a devastating blow to our renewed ambitions to eliminate malaria.
